# Factor analysis of the clustering of common somatic symptoms: a preliminary study

**DOI:** 10.1186/1472-6963-10-160

**Published:** 2010-06-10

**Authors:** Chung-Huang Tsai

**Affiliations:** 1 Department of Family Medicine, Cheng Ching Hospital, No. 118 sec. 3 Chung-Kang RD. Taichung 407, Taiwan; 2 Graduate Institute of Biochemical Sciences and Technology, Chaoyang University of Technology, No.168, Jifong E. Rd., Wufong Township, Taichung County 41349, Taiwan; 3 Department of Health Business Administration, HungKuang University, No 34, Chung-Chie Rd, Sha Lu, Taichung, 443, Taiwan

## Abstract

**Background:**

Studies of outpatient department patients indicate that somatic discomforts such as headache, neck pain, chest pain, low back pain, and gastrointestinal discomfort are commonly found in patients with multiple complaints. Clustering of some symptoms has been found in common somatic symptom analyses. Because of the complexity involved in the diagnosis of patients with multiple complaints, the aim of this study is to identify and classify patterns of somatic symptoms in individuals assessed during a health examination.

**Methods:**

A total of 683 patients (437 males, 246 females) received a one-day physical examination and completed a structured survey during the period from May 2007 to April 2008. A physical symptoms interview was conducted, and medical and demographic data was collected.

**Results:**

Based on the factor analysis, 4 clusters of symptoms were identified: 1) pain symptoms, 2) cold symptoms, 3) cardiopulmonary symptoms, and 4) gastrointestinal symptoms. The distribution of symptoms differed between males and females. After varimax rotation of factor patterns, 4 extracted factors emerged. In males, the factors were 1) pain symptoms, 2) cold symptoms, 3) cardiopulmonary symptoms, and 4) gastrointestinal symptoms. In females, the factors were 1) pain symptoms, 2) cold symptoms, 3) cardiopulmonary symptoms, and 4) head and gastrointestinal symptoms.

**Conclusions:**

Four clusters of somatic symptoms emerged for both males and females; however, the predominant symptoms were different in males and females. Females displayed more head-related symptoms than males. Patients should be thoroughly interviewed about additional symptoms within the same cluster after the recognition of a single somatic complaint.

## Background

In studies comparing Outpatient Department (OPD) patients with the general population, somatic discomforts such as dizziness/headache, neck pain, chest discomfort/chest pain, shortness of breath, low back pain, and gastrointestinal (GI) discomfort were commonly found in patients with multiple complaints [1-6]. The clustering of some symptoms has been historically found in common somatic symptom analyses. Blackwell et al. [7] and David et al. [8] divided common symptoms into 5 major categories: gastroenterological, pain, cardiopulmonary, neurological, and reproductive. Tsai et al. [9] separated the symptoms into two key categories, pain and cardiopulmonary. Other studies have highlighted gender differences in somatic symptoms of depression [10]. Crook et al. [1] found that there were similar characteristics for pain at different sites, and that some physical illnesses are associated with a clustering of somatic symptoms. A study of an ambulatory elderly population found that patients with multiple complaints tended to have depression, and that multiple somatic symptoms are a good indicator for depression in the elderly [11].

Somatic discomforts or pains [12] caused by cancer [13] and other incurable chronic diseases often result in complications such as depression. In addition, depression often exacerbates the discomfort and pain [12]. Some studies have shown that depressive and anxiety disorders are associated with pain, palpitations, dizziness, and nausea, [14-18] which suggests that physical and mental disorders are intertwined. When dealing with somatic symptoms, not only should physical diseases be taken into consideration, but also mental illness should be considered. Thus, it is especially important for family physicians to understand the clustering of common somatic symptoms so that they will not be overwhelmed when dealing with the complexity of multiple complaint patients [19].

Because of the complexity involved in the diagnosis of patients with multiple complaints, the aim of this study is to identify and categorize clustering of somatic symptoms such that if a patient has one somatic symptom, other symptoms in the same cluster may be investigated in order to improve the quality and accuracy of diagnosis in a primary care setting. We believe this descriptive analysis of common somatic symptoms may provide a reference point for medical personnel to use when evaluating patients with multiple complaints.

## Methods

### Participants

Our study included 683 individuals who received one-day physical examinations from May 2007 to April 2008 at a regional hospital in Taichung City. The study sample was a convenience sample. The one-day physical examination is a very popular health care program in Taiwan that includes a physical examination, laboratory tests, ultrasound examination, and endoscopic examination. Physical examinations were performed by physicians of various specialties, and physicians of Family Practice Department reviewed and interpreted the final report and made health-related suggestions to the patients.

The 683 individuals completed the physical examination, associated testing, and completed interviews that recorded demographic and symptom information. Patients who were seen but could not complete the examinations or interview/survey were not considered for inclusion in the analysis. The medical ethics committee at the Cheng-Ching Hospital approved the study in May 2007. Informed consent was obtained from all participants before their participation.

### Study design and methods

The symptom interview schedule was designed based on a Chinese revision of the Psychiatric Diagnostic Interview Schedule (DIS-CM) [20] that incorporated a common physical symptoms diagnostic table, foreign literature [21-23], and the study by Tsai et al. [9]. In order to find a common basis for all of the subjects, we tested 50 physical examinees. A total of 22 somatic symptoms commonly encountered during a physical examination were defined, i.e., neck pain and rigidity, lower back pain, partial somatic numbness and tingling, light-headedness, hand and foot arthralgia, muscle soreness, chest discomfort/chest pain, palpitations, poor appetite, GI discomfort, constipation/diarrhea, dizziness, headache, tinnitus, foreign bodily sensations and obstructive sensations in the throat, muscle weakness, hyperhidrosis, chill, fever, shivering, frequent micturition/nocturia (≥3 times per night), and skin itchiness. The internal consistency reliability (Cronbach's α) of the diagnostic schedule was 0.86.

Symptoms were graded on a 5-grade scoring system; 0 represented no symptoms, and 1, 2, 3, and 4 designated mild, intermediate, severe, and very severe symptoms, respectively. Patients completed the interview/survey on the day of the physical examination prior to discussion of the results with the attending physicians. The data were input and processed in a database using the Microsoft Excel 2003.

### Statistical analysis

Categorical data were presented as a number (%), and the Chi-square test was used to test the difference between genders. The level of significance was defined as 0.05. To reduce 22 symptoms into a smaller number of interpretable factors, exploratory factor analysis was performed.

The exploratory factor analysis was composed of 5 processes. To check the assumptions of the factor analysis, correlations among variables were examined initially. Twenty symptoms had a Pearson's correlation coefficient >0.3, which indicated a weak correlation between any two variables. The sampling adequacy was confirmed by an anti-image correlation matrix, the factorability was proved by a Kaiser-Meyer-Olkin index that was >0.6, and the result of Bartlett's test of sphericity indicated no identity matrix was present. Principal component analysis was used to extract summary factors, and only the factors with eigenvalues ≥1 were extracted. The varimax rotation method was performed to simplify the interpretations of summary factors; one symptom was considered to be loaded on a factor if its factor loading was ≥0.50. Finally, the name of a summary factor was denominated according to the loaded symptoms on a specific factor. All statistics were calculated with SPSS version 15.0 (SPSS Inc., Chicago, IL, USA).

## Results

A total of 817 subjects received the one-day physical exam. Among them, 134 participants (16.4%) were not included due to refusing to participate in the questionnaire or because of illiteracy. Thus, a total of 683 subjects were included in the study, 437 males and 246 females. Patient demographic data is presented in Table 1. More than half of the subjects (53.7%) were between 45 and 64 years of age. A majority of the subjects were married (81.0%), employed (76.6%), and had an advanced education level (college or above) (42.8%). When compared to females, more males were between 45 and 64 years of age (57.2% vs. 47.5%, *P *= 0.022), employed (89.2% vs. 54.1%, *P *< 0.001), had a higher education level (48.9% vs. 32.2%, *P *< 0.001), were smokers (40.5% vs. 6.1%, *P *< 0.001), and consumed alcohol (47.8% vs. 14.2%, *P *< 0.001).

**Table 1:** Patient characteristics

	Male (n = 437)	Female (n = 246)	*P*-value^1^
Age (years)			0.022*
< 45	161 (36.8)	104 (42.3)	
45-65	250 (57.2)	117 (47.5)	
≥65	26 (6.0)	25 (10.2)	
Education^2^			< 0.001*
Junior high school or below	95 (21.9)	98 (40.0)	
Senior high school	127 (29.2)	68 (27.8)	
College or above	213 (48.9)	79 (32.2)	
Religion			0.008*
None	76 (17.4)	40 (16.3)	
Folk belief	142 (32.5)	66 (26.8)	
Taoism	75 (17.1)	38 (15.4)	
Buddhism	131 (30.0)	79 (32.1)	
Others	13 (3.0)	23 (9.4)	
Marital status			< 0.001*
Single	43 (9.8)	20 (8.1)	
Married	378 (86.5)	175 (71.2)	
Other	16 (3.7)	51 (20.7)	
Employment			< 0.001*
Employed	390 (89.2)	133 (54.1)	
Unemployed	47 (10.8)	113 (45.9)	
Smokers	177 (40.5)	15 (6.1)	< 0.001*
Alcohol drinkers	209 (47.8)	35 (14.2)	< 0.001*

Data presented as number (percent).

^1^*P*-values were derived from the chi-square test.

^2^Missing data was noted.

*Indicates statistical significance, *P *< 0.05.

Table 2 presents the distribution of symptoms between males and females. Except for tinnitus and chest pain, which showed similar percentage of agreement among the genders, 20 out of 22 symptoms were more prevalent in females, and 10 out of those 20 symptoms, including dizziness (8.1%), headache (8.1%), sensation of dyspnea or asphyxia (20.4%), palpitations (6.0%), poor appetite (5.7%), stomach ache or abdominal discomfort (11.0%), constipation or diarrhea (9.7%), low back pain (12.6%), sensation of anesthesia (11.0%), sensation of heavy limbs (8.6%), joint pain (9.8%), feeling too warm or cold (2.8%), and frequent micturition (8.9%) exhibited a statistically significant difference between males and females (all, *P *< 0.05).

**Table 2:** Symptom distribution by gender

	Male (n = 437)	Female (n = 246)	*P-*value^1^
Dizziness	14 (3.2)	20 (8.1)	< 0.001*
Headache	13 (3.0)	20 (8.1)	< 0.001*
Tinnitus	24 (5.5)	13 (5.3)	0.850
Lump in throat	22 (5.0)	21 (8.5)	0.229
Neck pain or stiffness	45 (10.3)	42 (17.1)	0.061
Chest pain	21 (4.8)	12 (4.9)	0.063
Sensation of dyspnea or asphyxia	8 (1.8)	6 (20.4)	0.410
Palpitation	12 (2.8)	15 (6.0)	0.044*
Poor appetite	10 (2.3)	14 (5.7)	0.021*
Stomach ache or abdominal discomfort	25 (5.7)	27 (11.0)	0.031*
Constipation or diarrhea	16 (3.6)	24 (9.7)	0.001*
Low back pain	44 (11.1)	31 (12.6)	0.013*
Sensation of anesthesia	26 (6.0)	27 (11.0)	0.047*
Sensation of heavy limbs	9 (2.1)	21 (8.6)	< 0.001*
Joint pain	23 (5.2)	24 (9.8)	0.004*
Muscle weakness	17 (3.9)	17 (6.9)	0.155
Itchiness	15 (3.5)	12 (4.9)	0.478
Cold sweating	9 (2.1)	9 (3.6)	0.705
Feeling too warm or cold	1 (0.2)	7 (2.8)	< 0.001*
Shuddering	2 (0.4)	4 (1.6)	0.422
Muscle pain	25 (5.7)	23 (9.4)	0.115
Frequent micturition	14 (3.2)	22 (8.9)	0.009*

Data presented as number (percent).

Note: Severe and very severe symptoms were combined due to the sample size in each category.

^1^Chi-square test was used.

*Significantly different between genders, *P *< 0.05.

There were 4 factors that explained 59.1% of the total variance in the 683 subjects (Table 3). Factor 1 (pain symptoms) included neck pain, low back pain, sensation of anesthesia, sensation of heavy limbs, joint pain, muscle weakness and muscle pain, and these comprised the largest proportion (20.9%) of the total variance. Factor 2 (cold symptoms) included cold sweating, feeling too warm or cold, shudder, and dizziness and contributed 15.2% of the total variance. Factor 3 (cardiopulmonary symptoms) included chest pain, sensation of dyspnea or asphyxia, and palpitations, and contributed 11.8% of the total variance. Factor 4 (GI symptoms) included poor appetite, stomach pain, and constipation or diarrhea, and accounted for 11.2% of the total variance. The Cronbach's α coefficients for Factor 1, 2, 3, and 4 were 0.861, 0.682, 0.748, and 0.651, respectively.

**Table 3:** Factor loading of varimax rotated factor pattern for 683 subjects

	Factor			
	
	1 Pain Symptoms	2 Cold Symptoms	3 Cardiopulmonary Symptoms	4 Gastrointestinal Symptoms
Dizziness	0.28	**0.51**	0.25	0.24
Neck pain or stiffness	**0.61**	0.08	0.28	0.23
Chest pain	0.28	0.13	**0.79**	0.13
Sensation of dyspnea or asphyxia	0.22	0.20	**0.76**	0.08
Palpitation	0.24	0.37	**0.59**	0.27
Poor appetite	0.18	0.29	0.11	**0.65**
Stomach ache or abdominal discomfort	0.14	0.20	0.26	**0.69**
Constipation or diarrhea	0.22	0.05	0.03	**0.77**
Low back pain	**0.69**	0.06	0.24	0.24
Sensation of anesthesia	**0.74**	0.21	0.08	0.10
Sensation of heavy limbs	**0.61**	0.46	0.11	0.11
Joint pain	**0.72**	0.23	0.12	0.06
Muscle weakness	**0.55**	0.45	0.16	0.15
Cold sweating	0.12	**0.71**	0.07	0.07
Feeling too warm or cold	0.12	**0.77**	0.12	0.18
Shuddering	0.13	**0.61**	0.18	0.11
Muscle pain	**0.75**	0.03	0.23	0.21
% Variance explained	20.9	15.2	11.8	11.2
Cumulative variance	20.9	36.1	47.9	59.1

Factor loadings ≥0.50 are in bold.

Because the distribution of symptoms for males differed significantly from that of females, further analysis by gender was performed to check whether the summary factors of both genders were different. The results presented in Table 4 and Table 5 indicated both genders exhibit 4 extracted factors. Among them, 3 common factors for both genders, pain symptoms (Factor 1), cold symptoms (Factor 2) and Factor 3 (cardiopulmonary symptoms), were found. The pain symptoms contributed 19.1% of the variance for males. For females, symptoms of exhaustion accounted for 20.1% of the variance (frequent micturition was included in this group). Regardless of gender, 3 cold symptoms were loaded on the factor called cold symptoms, which explained the 13.3% and 12.2% of the variance in males and females, respectively. Factor 3 consisted of chest pain, sensation of dyspnea or asphyxia, palpitation and frequent micturition; which explained 12.6% and 12.7% of variances in males and females. The frequent micturition was included in Factor 3 for males but it was an important component of cold symptoms for females. The Cronbach's α coefficient for Factor 1, Factor 2 and Factor 3 were 0.844, 0.593 and 0.698 for males, and 0.870, 0.699 and 0.749 for females, respectively.

**Table 4:** Factor loading of varimax rotated factor pattern for 437 males

	Factor			
	
	1 Pain Symptoms	2 Cold Symptoms	3 Cardiopulmonary Symptoms	4 Gastrointestinal Symptoms
Neck pain or stiffness	**0.61**	-0.16	0.22	0.44
Chest pain	0.24	0.03	**0.74**	0.28
Sensation of dyspnea or asphyxia	0.22	0.13	**0.70**	0.17
Palpitation	0.18	0.35	**0.58**	0.32
Poor appetite	0.17	0.36	0.12	**0.62**
Stomach ache or abdominal discomfort	0.15	0.14	0.14	**0.76**
Constipation or diarrhea	0.16	0.27	0.07	**0.65**
Low back pain	**0.65**	-0.04	0.23	0.32
Sensation of anesthesia	**0.72**	0.21	0.19	0.07
Sensation of heavy limbs	**0.55**	0.45	0.24	0.04
Joint pain	**0.72**	0.31	0.03	0.04
Muscle weakness	**0.53**	0.48	0.29	0.07
Clod sweating	0.09	**0.59**	0.19	0.23
Feeling too warm or cold	0.03	**0.71**	0.13	0.21
Shuddering	0.23	**0.63**	0.09	0.13
Muscle pain	**0.74**	0.11	0.17	0.22
Frequent micturition	0.10	0.20	**0.60**	-0.10
% Variance explained	19.1	13.3	12.6	12.2
Cumulative variance	19.1	32.4	45.0	57.1

Factor loadings ≥0.50 are in bold.

**Table 5:** Factor loading of varimax rotated factor pattern for 246 females

	Factor			
	
	1 Pain Symptoms	2 Cold Symptoms	3 Cardiopulmonary Symptoms	4 Head and Gastrointestinal Symptoms
Dizziness	0.20	0.44	0.04	**0.63**
Headache	0.15	0.30	0.09	**0.64**
Tinnitus	0.22	0.13	-0.01	**0.62**
Neck pain or stiffness	**0.57**	0.16	0.25	0.30
Chest pain	0.29	0.13	**0.71**	0.12
Sensation of dyspnea or asphyxia	0.23	0.23	**0.74**	0.00
Palpitation	0.23	0.24	**0.58**	0.39
Stomach ache or abdominal discomfort	0.08	0.09	0.47	**0.60**
Constipation or diarrhea	0.22	-0.30	0.22	**0.54**
Low back pain	**0.66**	0.00	0.32	0.28
Sensation of anesthesia	**0.75**	0.07	0.09	0.22
Sensation of heavy limbs	**0.66**	0.35	0.19	0.19
Joint pain	**0.64**	0.01	0.36	0.21
Muscle weakness	**0.63**	0.41	0.12	0.15
Cold sweating	0.19	**0.75**	0.10	0.08
Feeling too warm or cold	0.17	**0.67**	0.24	0.35
Shuddering	0.06	**0.56**	0.37	0.13
Muscle pain	**0.70**	-0.09	0.44	0.10
Frequent micturition	**0.58**	0.35	-0.06	-0.02
% Variance explained	20.1	12.2	12.7	13.1
Cumulative variance	20.1	58.0	45.9	33.1

Factor loadings ≥0.50 are in bold.

The other summary factors for males were Factor 4 (GI symptoms) which accounted for 12.2% of the variance (Cronbach's α = 0.675). Factor 4 consisted of all 3 digestive symptoms (Table 4). For females, Factor 4 (head and GI symptoms) consisted of head symptoms (dizziness, headache, and tinnitus) and GI symptoms (stomach pain and constipation or diarrhea). In the cluster, poor appetite was not included in female group. In females, 13.1% variance explained by head and GI symptoms (Cronbach's α = 0.699) (Table 5).

## Discussion

The aim of this study was to identify and categorize clustering of symptoms such that if a patient has one somatic symptom, other symptoms in the same cluster may be investigated in order to improve the quality and accuracy of diagnosis a primary care setting. The clustering of some symptoms suggests that when patients have one somatic symptom, other symptoms in the same cluster should also be evaluated. Based on the factor analysis, we identified 4 clusters of symptoms: Factor 1 was related to pain, Factor 2 was associated with cold-like symptoms, Factor 3 involved symptoms associated with heart and respiration, and Factor 4 was associated with the GI system. The 4 factors categorized 17 out of 22 symptoms. Pain symptoms were the highest in terms of the number of symptoms, percentage of variance explained, and prevalence.

With the exception of differences in a few symptoms, the 4 factors described were comparable to those identified in other foreign and domestic studies [8,9,24]. David et al. [8] classified the common symptoms into 5 major categories, and 3 of them were very similar to the findings in our study. The 3 major categories similar to ours are Category 1, GI symptoms (nausea, vomiting, and abdominal pain); Category 2, pain symptoms (generalized pain, limb pain, back pain, joint pain, dysuria, and headache); and Category 3, cardiopulmonary symptoms (difficulty in breathing, palpitations, chest pain, and dizziness) [8]. Other studies have indicated that females tend to have headache and abdominal pain symptoms with more frequency than males [1,25]. Our results are consistent with previous studies in that 20 out of 22 symptoms were more prevalent in females, and there was a statistical significance between males and females in 10 out of those 20 symptoms.

With regard to pain symptoms in general, except for headache, individuals older than 65 years experienced more pain symptoms than any other age group. Among the pain symptoms, lower back pain and neck pain and rigidity had the highest prevalence, which is consistent with other studies on seniors [25]. Chest pain was the fifth most common symptom at any age, while other pain symptoms were variously distributed in different age ranges. For example, individuals who were ≥45 years old more commonly experienced hand and foot arthralgia as compared to younger people. On the other hand, individuals who were < 65 years of age had a greater incidence of muscle pain. Other studies have also indicated that sites of pain were dissimilar between seniors and adults, with seniors having fewer headaches and abdominal pain [2].

Other work indicated dizziness was the third commonly experienced symptom found in a senior primary care setting [3]. Tsai et al. [9] also found that it was fifth out of the 15 most common symptoms found in seniors >70 years old [9]. In our study, it was the fourth most common symptom. According to Furman et al. [4], there were large variations from study to study, ranging from 20% to 50%; however all revealed a high prevalence [4].

A study of 14 common symptoms in 1000 patients in an internal medicine department found that 38% of patients had two or more symptoms [5]. A study by Tsai et al. [9] that targeted an elderly population also indicated that 76.49% of the patients had 2 or more symptoms. In our work, we found that 87.5% of the patients had two or more symptoms. Some experts have suggested that it is not important to assign a diagnosis of somatoform disorder as long as patient discomforts can be resolved [8]. Furthermore, the combinations of symptoms in this study represent a clustering phenomenon of common symptoms, which may be found in both physical and mental illness.

There are limitations to this study that should be considered. The data are only from a single medical center in central Taiwan, and thus the results may not be applicable to other patient populations. Also, the study did not include a measure for depression and anxiety. That measure was beyond the scope of this work, and we consider this study a preliminary analysis to guide further research. The critical characteristics of frequency and abundance of symptoms were beyond our objective in this study; however, the severity of the disease may affect the structure of clustering, an analysis which should be included in future study.

## Conclusions

We used factor analysis to evaluate 22 common symptoms and created 4 symptom categories seen in primary care patients. The clustering of some somatic symptoms suggests that when patients have one somatic symptom, other symptoms in the same cluster should also be evaluated. We believe this descriptive analysis of common somatic symptoms may provide a reference point for medical personnel to use when evaluating patients with multiple complaints.

## Abbreviations

DIS-CM: Chinese Revision of the Psychiatric Diagnostic Interview Schedule; DSM-IV: The Diagnostic and Statistical Manual of Mental Disorders; Fourth Edition; OPD: Outpatient Department.

## Competing interests

The author declares that they have no competing interests.

## Authors' contributions

CHT designed the study, participated in data collation, statistical analysis, and writing the draft of the manuscript. The author read and approved the final manuscript.

## Pre-publication history

The pre-publication history for this paper can be accessed here:

http://www.biomedcentral.com/1472-6963/10/160/prepub
